# Genome-wide association study for T lymphocyte subpopulations in swine

**DOI:** 10.1186/1471-2164-13-488

**Published:** 2012-09-18

**Authors:** Xin Lu, Wei-Xuan Fu, Yan-Ru Luo, Xiang-Dong Ding, Jia-Peng Zhou, Yang Liu, Jian-Feng Liu, Qin Zhang

**Affiliations:** 1 Key Laboratory Animal Genetics, Breeding and Reproduction, Ministry of Agriculture, College of Animal Science and Technology, National Engineering Laboratory for Animal Breeding, China Agricultural University, Beijing, 100193, China; 2 State Key Laboratory for Infectious Disease Prevention and Control, National Institute for Communicable Disease Control and Prevention, Chinese Center for Disease Control and Prevention, P.O. Box 5, Chang ping, Beijing, 102206, China; 3 Department of Animal and Food Sciences, University of Delaware, Newark, DE, 19716, USA

**Keywords:** T lymphocyte subpopulations, Genome-wide association study, Swine

## Abstract

**Background:**

Lymphocytes act as a major component of the adaptive immune system, taking very crucial responsibility for immunity. Differences in proportions of T-cell subpopulations in peripheral blood among individuals under same conditions provide evidence of genetic control on these traits, but little is known about the genetic mechanism of them, especially in swine. Identification of the genetic control on these variants may help the genetic improvement of immune capacity through selection.

**Results:**

To identify genomic regions responsible for these immune traits in swine, a genome-wide association study was conducted. A total of 675 pigs of three breeds were involved in the study. At 21 days of age, all individuals were vaccinated with modified live classical swine fever vaccine. Blood samples were collected when the piglets were 20 and 35 days of age, respectively. Seven traits, including the proportions of CD4+, CD8+, CD4+CD8+, CD4+CD8−, CD4−CD8+, CD4−CD8− and the ratio of CD4+ to CD8+ T cells were measured at the two ages. All the samples were genotyped for 62,163 single nucleotide polymorphisms (SNP) using the Illumina porcineSNP60k BeadChip. 40833 SNPs were selected after quality control for association tests between SNPs and each immune trait considered based on a single-locus regression model. To tackle the issue of multiple testing in GWAS, 10,000 permutations were performed to determine the chromosome-wise and genome-wise significance levels of association tests. In total, 61 SNPs with chromosome-wise significance level and 3 SNPs with genome-wise significance level were identified. 27 significant SNPs were located within the immune-related QTL regions reported in previous studies. Furthermore, several significant SNPs fell into the regions harboring known immunity-related genes, 14 of them fell into the regions which harbor some known T cell-related genes.

**Conclusions:**

Our study demonstrated that genome-wide association studies would be a feasible way for revealing the potential genetics variants affecting T-cell subpopulations. Results herein lay a preliminary foundation for further identifying the causal mutations underlying swine immune capacity in follow-up studies.

## Background

Infectious diseases cause many serious economic and welfare problems in current swine industry. Some of porcine diseases belong to zoonoses, and put people at risk to infections. Even though there are many ways, such as vaccination injection, antibiotic treatment and isolation, to deal with issue of diseases, infectious diseases could not be solved completely [1]. On the other hand, genetics and breeding are working on improving immune capacity of animals through artificial selection, which maybe provides a promising strategy. Moreover, swine is increasingly used as a large animal model for several human diseases [2-4]. The porcine immune system is becoming more and more interesting in the field of both basic and applied research.

The immune system plays an essential role in disease resistance of animals. Lymphocytes have been widely recognized as a major component of the adaptive immune system, bearing very crucial responsibility for immunity. Lymphocytes are basically divided into two categories, namely T and B lymphocytes, each responsible for a particular branch of the immune system. T-lymphocytes (T-cells) are mostly responsible for fighting microbes, antigens or foreign substances inside the cells, triggering cell-mediated immunity. The proportions of T-cell subpopulations in peripheral blood vary with health and disease status. CD4 (an antigenic marker of helper T-cell) binds to relatively invariant sites on class II major histocompatibility complex (MHC) molecules outside the peptide-binding groove, which interacts with the T-cell receptor (TCR) [5,6]. The functions of CD4 are to initiate or augment the early phase of T-cell activation. CD4+ T cells are responsible for activating and directing other immune cells. They are essential in determining B cell antibody class switching, activating cytotoxic T cells, and maximizing bactericidal activity of phagocytes such as macrophages. Shedlock and Shen [7] showed that CD4+ cells are required in the priming phase for functional CD8 memory. CD8 antigen is a cell surface glycoprotein found in most cytotoxic T-cells that mediates efficient cell-cell interactions within the immune system. CD8 antigen, together with other T-cell receptors on T-cells, recognizes antigen processed by antigen presenting cells (APCs) in the context of class I MHC molecules [8].

In addition to the individual functions of CD4 and CD8, different combinations of them, *i.e.*, CD4+CD8+, CD4+CD8−, CD4−CD8+ and CD4−CD8−, as well as the ratio of CD4+ to CD8+ also vary with health and disease status, and thus are highly relevant to immune capability of individuals. CD4-CD8+ are MHC class I restricted and mainly recognize replicating viral antigens. CD4+CD8- are MHC class II restricted and respond to nonreplicating protein antigens processed by APCs [9,10]. Differences in ratios of CD4 to CD8 are MHC haplotype-dependent [11].

Immunology is a fast growing research area. Gerner *et al*. summarized the current knowledge about porcine T lymphocytes and porcine T-lymphocyte subpopulations [12]. Lymphocytes expressing markers CD4 or CD8 alone and CD4 and CD8 together are important in viral clearance by secreting IFN-γ and mediating pathogen specific cytotoxicity [13]. Classical Swine Fever Virus (CSFV)-specific T-cell epitopes, the epitope peptide 290, harbors a CSFV-specific helper T-cell epitope and a CTL epitope, which could elicit both CD4+ and CD8+ T-cell responses [14]. Previous studies have shown that production of CSFV-specific CD8+ CTLs represents an important defense mechanism in the elimination of cells infected by CSFV [15]. Antigen specific lysis of CSFV-infected targets was found to be performed by CD4+ T cells. It has previously been demonstrated that CD8+ T cells lysed pseudorabies virus (PRV)-infected cells [16] and CD4+ producing T cells play important roles in conferring protection against a lethal PRV infection [17]. The immune system of swine differs markedly from that of humans and mice. Swine has a substantial number of CD4-CD8-T lymphocytes in peripheral blood [10,18-20]. Swine is also the only species so far known to hold a substantial number of resting extrathymic CD4+CD8+ T-cells [10,20-22]. Summerfield *et al.*[23] demonstrated that CD4+CD8+ cells can function as memory T-helper cells which proliferate upon stimulation with recall antigen. The CD4+CD8+ T cells were found to be associated with protection in pigs vaccinated against PRV [24]. The study of the cellular immune response to virus or vaccines in the natural host is of the utmost importance for understanding the interaction between the pathogen and the swine immune system [25].

Differences in proportions of T-cell subpopulations in peripheral blood among individuals under same conditions provide evidence of genetic control on these traits, but little is known about the genetic mechanism of them, especially in swine. Identification of the genetic control on these variants may help the genetic improvement of immune capacity in animals through selection. So far only two reports focused on QTL mapping for T-cell subpopulations in peripheral blood in pig [26,27]. QTL mapping has been very successful in domestic animals but the identification of quantitative trait mutations (QTMs) is still a challenge although a few prominent successful cases have been reported [28].

Recently, the first high-density 60K porcine SNP array has been developed [29], which offers the prerequisite for genome-wide association study (GWAS), a powerful approach for high-resolution mapping of loci controlling complex traits in domestic animals. Duijvesteijn et al. [30] firstly reported a GWAS for androstenone levels in pigs by 60K SNP array and revealed a cluster of candidate genes on *Sus scrofa* chromosome (SSC) 6. More recently, Fan *et al.*[31] preformed a GWAS for body composition and structural soundness traits of pigs and identified several genes by functional clustering analyses. Up to now, GWAS have been becoming a most commonly-used strategy for gene identification for complex traits in animals as well as humans.

In this study, we performed GWAS for T lymphocyte traits in swine by genotyping 675 pigs from 3 breeds (including a Chinese indigenous breed) based on the 60K SNP array, with measuring seven phenotypes of T-cell subpopulations in 562 piglets. A suite of significant SNPs associated with T-cell subpopulations at either the genome-wise or chromosome-wise were identified. These promising loci may be considered as preliminary foundation for further replication studies and eventually unraveling the causal mutations for T-cell subpopulations traits in swine.

## Methods

### Animal resource

The animal resource used in this study consists of 675 pigs of three breeds (Landrace, Yorkshire and Songliao Black pigs) including 562 piglets and their parents. The structure of experimental population was given in Table 1. All individuals were raised under standard indoor conditions at the experimental farm of the Institute of Animal Sciences, Chinese Academy of Agricultural Sciences, Beijing, China. At 21 days of age, all piglets were vaccinated with 4 doses live Classical Swine Fever Virus (CSF) Vaccine (Rabbit origin, tissue virus ≥ 0.01 mg/dose) (Qilu Animal Health Products Co., Ltd., Shandong, China) through intramuscular injection. Blood samples were collected from the jugular vein of each piglet one day before the vaccination (day 20), and two weeks after the vaccination (day 35), respectively. And blood samples were directly injected into eppendorf tubes containing 60 μl of 20% EDTA in phosphate-buffered saline (PBS). In addition, ear tissues of all individuals were also collected. The whole procedure for collection of the samples (blood and ear tissue) was carried out in strict accordance with the protocol approved by the Animal Welfare Committee of China Agricultural University (Permit number: DK997).

**Table 1:** Animal resource for GWAS

**Breed**	**Sires**	**Dams**	**Piglets**	**Total**
Landrace	4	13	68	85
Yorkshire	16	63	415	494
Songliao Black	3	14	79	96
Total	23	90	562^1^	675^2^

Note 1: The total number of the piglets which were phenotyped 2: The total number of all the animals (including piglets and their parents) which were genotyped.

### Measurement of phenotypes

For all individuals, seven different types of phenotypes of T-cell subpopulations, including the proportions of CD4+, CD8+, CD4+CD8+, CD4+CD8−, CD4−CD8+, CD4−CD8− and the ratio of CD4+ to CD8+ T cells, were obtained by the double cytofluorometric analysis. The blood cells were incubated with 10 μl of mouse anti porcine CD4-FITC (Serotec UK) and 10 μl of mouse anti porcine CD8-RPE (Serotec UK) for 30 min, and then washed with 0.1 M PBS (pH 7.2, containing 0.3% bovine serum albumin). The red blood cells were digested by 0.1% ammonium oxalate solution. The stained cells were detected by EPICS Flow Cytometer (Beckman-Coulter Company, USA).

### Genotyping

DNA was extracted from ear tissue samples of all pigs, including piglets and their parents and genotyped using the Illumina PorcineSNP60K BeadChip containing 62,163 SNPs. Features of the Illumina PorcineSNP60K BeadChip have been detailed previously [29]. All individuals were genotyped using BeadStudio (Illumina) in terms of a custom cluster file developed from the 675 samples investigated.

### Genotype quality control

To assess the technical reliability of the genotyping panel, a randomly selected DNA sample was genotyped twice and over 99% identity of called genotypes (two mismatches) was obtained. This demonstrates the technically robust feature of the 60K SNP BeadChip panel employed herein. All the samples included are with a minimum of 95% call rate.

We performed a 2-step quality control procedure for all genotyped piglets as follows. In first quality control, we discards meaningless SNPs (minor allele frequency (MAF) = 0 or call rate = 0) out of the initial full–set of 62,163 SNPs for each breed. So 8307/6258/4838 SNPs were moved out for Landrace, Yorkshire and Songliao Black respectively. Then BEAGLE software (Version 3.3.2) [32] was adopted to impute missing genotype for all the SNPs with assigned physical positions. In second quality control, out of the imputed SNPs, SNPs with a call rate < 90% (n = 286/276/428 for Landrace, Yorkshire and Songliao Black respectively) were discarded, and SNPs with MAF < 0.03 in the resource population (n = 3704/5887/7868 for Landrace, Yorkshire and Songliao Black respectively) were discarded, and SNPs with Hardy–Weinberg equilibrium (*p* < 10^-6^) (n = 171/602/220 for Landrace, Yorkshire and Songliao Black respectively) were discarded. In this way, 49,140/49,695/48,809 SNPs were available for Landrace, Yorkshire and Songliao Black respectively. Finally 40833 common SNPs in three breeds were selected for the subsequent analyses. The distribution of common SNPs across genome after filtering was presented in Additional file 1: Table S1.

### Statistical analyses

#### Parentage test

Considering the probability of potential parentage mistakes in the original pedigree records, we adopted Cervus [33] to estimate the most possible parent-offspring pair with maximum likelihood method using 100 randomly chosen autosomal SNPs with 100% call rate. Eventually, about 10% piglets had incorrect recorded sires or dams. Among these individuals with pedigree errors, 64% of them were reassigned to correct parents based on the information of SNP genotypes, and the corrected pedigree information were used in the subsequent analyses, while the remaining individuals which could not be referred to true parents were treated as offspring with unknown parents.

#### Mixed model based single locus regression analyses (MMRA)

Similar to our previous study by Jiang *et al.*[34], we performed association tests for each SNP via regression analysis based on the following linear mixed model:

(1) y=1μ+kc+Mf+Tv+bX+Za+e

where **y** is the vector of phenotypes of all piglets on Day 35; ***μ*** is the overall mean; **c** is the vector of phenotypes of all piglets on Day 20, *k* is the regression coefficient of the phenotypic observations on day 35 on those on day 20; **f** is the vector of the fixed effects, including effect of breed and batch of sampling, M is the incidence matrix of **f**; **v** is the vector of random litter effects, including effect of different litters, **T** is the incidence matrix of **v**; **X** is the vector of the SNP genotype indicators which take values 0, 1 or 2 corresponding to the three genotypes 11, 12 and 22 (assuming 2 is the allele with a minor frequency), *b* is the regression coefficient of phenotypes on day 35 on SNP genotypes. **a** is the vector of the residual polygenic effect with $a\sim N(0,A\sigma_{a}^{2})$ (where **A** is the additive genetic relationship matrix and is the additive variance), **Z** is the incidence matrix of **a**; **e** is the vector of residual errors with $e\sim N(0,I\sigma_{e}^{2})$ (where $\sigma_{e}^{2}$ is the residual error variance). For each SNP, the estimate of *b* and the corresponding sampling variances $Var(\overset{\wedge }{b})$ can be obtained via mixed model equations (MME), and a Wald Chi-squared statistic ${\overset{\wedge }{b}}^{2}/Var(\overset{\wedge }{b})$ with df = 1 is constructed to examine whether the SNP is associated with the trait surveyed.

We employed Fortran 95 to code the computing program for the method and it is available upon request.

#### Statistical Inference

For the analyses above, the permutation method was adopted to adjust for multiple testing for the number of SNP loci detected. In our method, the observations of each phenotype were randomly shuffled 10,000 times and the empirical critical value was determined by choosing the 95th percentile of the highest statistic over the 10,000 permutation replicates at both genome-wise and chromosome-wise. We declared a significant SNP at a 0.05 significance level if its raw value of the Wald Chi-squared statistic was larger than the empirical critical value.

#### Population stratification assessment

Confounding due to population stratification has been considered as a major plague to the validity of genetic association studies [35]. To view if the population stratification exists in our experimental population, we examined the distribution of the test statistics obtained from the numerous association tests performed and assessed their deviation from the expected distribution of no SNP being associated with the trait of interest by utilizing a quantile-quantile (Q-Q) plot, which is a routine and most frequently used tool for scrutinizing the population stratification in GWAS.

#### Linkage disequilibrium block analyses

Linkage disequilibrium (LD) block analyses were performed for the chromosomal regions with multiple significant SNPs clustered around genome-wise significant SNPs. The LD blocks were defined using Haploview (Version 4.2) [36], and the LD blocks were defined by the criteria of Gabriel et al. [37] to further pinpoint potential candidate genes affecting T-cell subpopulations.

## Results

### Alterations of proportions of T-cell subpopulations in peripheral blood after challenge

The descriptive statistics of T-cell subpopulations or their ratio in peripheral blood on day 20 (the day before vaccinating) and day 35 (the day two weeks after vaccinating) are shown in Table 2.

**Table 2:** Descriptive statistics analysis and estimates of variance components of immune traits in piglets

**Trait**	**Test Day**	**Mean**	**Standard Deviation**	**CV(%)**	**Variance component of genetic effect**	**Variance component of litter effect**
CD4-CD8- T %	20	36.2^a^	25.98	71.76	32.65	33.28
	35	35.7 ^a^	10.77	30.18		
CD4+CD8+ T %	20	8.11 ^a^	3.70	45.63	3.43	3.54
	35	11.23^b^	5.06	45.07		
CD4+CD8- T %	20	19.87 ^a^	7.76	39.08	2.90	8.26
	35	16.38^b^	7.28	44.42		
CD4-CD8+ T %	20	36.8 ^a^	13.57	36.88	6.88	35.45
	35	36.77 ^a^	11.53	31.37		
CD4+ T %	20	27.92 ^a^	8.64	30.94	20.14	10.35
	35	27.64 ^a^	8.83	31.96		
CD8+ T %	20	44.93 ^a^	14.14	31.47	2.44	48.44
	35	47.96^b^	12.57	26.22		
CD4+ / CD8+	20	0.72 ^a^	0.39	54.2	0.74E-02	0.81E-02
	35	0.63^b^	0.29	46.71		

Note: In the third column, different letters in superscript denote significant difference between ages 20 and 35 for the same trait.

Compared with the measurements on day 20, the proportion of CD4+CD8- T cells and the ratio of CD4+ to CD8+ in blood on day 35 decreased, while the proportion of CD4+CD8+ T cells and CD8+ T cells in blood at day 35 increased significantly. The proportion of CD4+, CD4-CD8- and CD4-CD8+ T cells on day 35 did not changed significantly.

### Significant SNPs

Both genome-wise significant SNPs and chromosome-wise significant SNPs for the seven traits are presented in Table 3. The profiles of *p* values (in terms of –log10*p* value) of all tested SNPs for the seven phenotypes of T-cell subpopulations are shown in Additional file 2: FigureS1. In total, 64 significant (P < 0.05) SNPs reached the chromosome-wise level. Among these identified SNPs, three reached the genome-wise significance level. In addition, some of these SNPs were identified to be associated with more than one trait, *e.g.*, ALGA0006170 on SSC1 (associated with CD8+ and CD4-CD8- T cells), H3GA0041012 on SSC14 (associated with the ratio of CD4+ to CD8+, CD4+CD8- and CD4-CD8+ T cells), ALGA0122248 on SSC16 (associated with CD4+ and the ratio of CD4+ to CD8+ T cells), INRA0055474 on SSC18 (associated with CD4+ and CD4+CD8- T cells), ALGA0098112 on SSC18 (associated with CD4+CD8- and the ratio of CD4+ to CD8+ T cells) and MARC0089391 on SSC18 (associated with CD4+CD8- and the ratio of CD4+ to CD8+ T cells). Several regions harbour more than one significant SNP, *e.g.*, 10 significant SNP in the 0.8 Mb region (form 6.6 Mb to 7.4 Mb) on SSC12 and 6 significant SNP in the 1.4 Mb region (form 116.7 Mb to 118.1 Mb) on SSC13.

**Table 3:** Significant SNPs for T-cell subpopulations

**Trait**	**No. SNPs**	**SNP Name**	**SSC.**	**Position**	**Significance level**^**b**^	**Nearest gene**	**Distance (bp)**
				**(bp)**^**a**^			
CD4-CD8- T%	3	ALGA0006170	SSC1	147676962	A	SELS	101263
		ALGA0027442	SSC4	106255554	A	LOC100515138	15207
		MARC0035862	SSC17	26683704	A	FLRT3	999166
CD4+CD8+ T%	15	ASGA0000475	SSC1	5393161	A	QKI	197563
		MARC0090836	SSC5	18263549	A	LOC100523435	8399
		H3GA0016197	SSC5	30846602	A	LOC100514286	within
		DRGA0005876	SSC5	52631994	A	LOC100153016	7905
		ASGA0093882	SSC8	27821389	A	LOC100515221	68339
		MARC0103793	SSC9	117134341	A	NCF2	within
		ALGA0061180	SSC11	18408471	A	LOC100518860	within
		ALGA0062506	SSC11	55628913	A	RNF219	152990
		DRGA0012994	SSC13	116721928	A	LOC100518275	Within
		ASGA0059251	SSC13	116794774	A	LOC100518275	within
		H3GA0037561	SSC13	117065279	A	LOC100518275	within
		MARC0024545	SSC13	117511306	A	LOC100739759	within
		H3GA0037568	SSC13	117963270	A	CLDN11	within
		ALGA0072642	SSC13	118134975	A	SLC7A14	22223
		ASGA0077977	SSC17	59215829	A	CBLN4	within
CD4+CD8- T%	31	SIRI0000967	SSC1	65386385	A	MAP3K7	93369
		ALGA0109882	SSC1	65402776	A	MAP3K7	76978
		ALGA0003935	SSC1	67256904	A	LOC100152346	within
		MARC0008049	SSC6	74003688	A	LOC100738715	within
		ALGA0105115	SSC9	127202082	A	CD46	12474
		MARC0076632	SSC9	127394064	B	LOC100514786	within
		ASGA0046812	SSC10	16205420	A	LOC100513811	within
		ALGA0057450	SSC10	16220722	A	LOC100513811	within
		ALGA0061535	SSC11	23576083	A	TRNAE-UUC	42913
		M1GA0015162	SSC11	61231196	A	LOC100154696	within
		ASGA0051093	SSC11	61450492	A	LOC100154696	112456
		ALGA0108362	SSC12	6596691	A	LOC100518214	225784
		MARC0009109	SSC12	6637944	A	SOX9	229926
		ALGA0106073	SSC12	6663787	A	SOX9	204083
		ASGA0052986	SSC12	6791202	A	SOX9	76668
		ASGA0052974	SSC12	6826155	A	SOX9	41715
		ALGA0064738	SSC12	6891741	A	SOX9	20500
		H3GA0033370	SSC12	6976387	B	LOC100518393	21514
		ASGA0053002	SSC12	7105246	A	LOC100519105	55408
		ASGA0104370	SSC12	7185885	A	LOC100519105	136047
		ALGA0064767	SSC12	7400177	A	LOC100519105	350339
		ALGA0078815	SSC14	76804400	A	LOC100521406	85124
		H3GA0041012	SSC14	77946383	A	PSAP	within
		ASGA0065687	SSC14	107127514	A	HTR7	260753
		ALGA0087956	SSC15	123078022	A	LOC100516232	22744
		INRA0055474	SSC18	21991584	A	LOC100622672	28674
		ALGA0097499	SSC18	22003396	A	LOC100513190	3075
		ASGA0079256	SSC18	22055859	A	LOC100525548	1899
		ALGA0098112	SSC18	38463920	A	BMPER	113622
		MARC0089391	SSC18	38488630	A	BMPER	88912
		MARC0024065	SSC18	50965324	A	LOC100622432	within
CD4-CD8+ T%	3	ASGA0022812	SSC4	126976845	A	LOC100737939	107579
		H3GA0041012	SSC14	77946383	A	PSAP	within
		ALGA0103427	SSC15	127742340	A	IKZF2	within
CD4+ T%	6	ASGA0023262	SSC4	130826499	A	LOC100151966	174827
		ALGA0030335	SSC5	7606208	A	LOC100512654	6682
		ALGA0122248	SSC16	73417692	A	LOC100518939	70693
		INRA0055474	SSC18	21991584	A	LOC100513190	within
		ASGA0079256	SSC18	22055859	A	LOC100525548	1899
		ASGA0080319	SSC18	49513089	A	LOC100516656	43287
CD8+ T%	1	ALGA0006170	SSC1	147676962	B	SELS	101263
CD4+:CD8+	5	H3GA0041012	SSC14	77946383	A	PSAP	within
		ALGA0117513	SSC15	123215784	A	LOC100518938	11678
		ALGA0122248	SSC16	73417692	A	LOC100738933	403285
		ALGA0098112	SSC18	38463920	A	BMPER	113622
		MARC0089391	SSC18	38488630	A	BMPER	88912

^a^: Derived from the recent porcine genome sequence assembly (Sscrofa9.2) ( http://www.ncbi.nlm.nih.gov/projects/mapview/map_search.cgi?taxid=9823&build=previous).

^b^: A means chromosome-wise significance level, B means genome-wise significance level.

### Population stratification assessment

**Figure 1:**
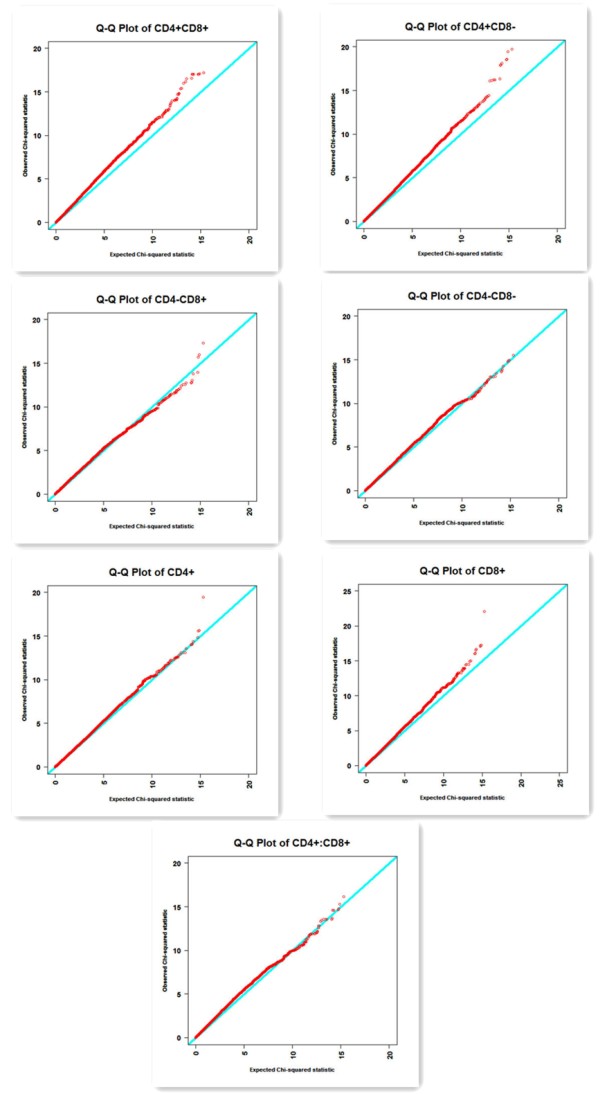
**Quantile-quantile (Q-Q) plots of test statistics in GWA for T-cell subpopulations.** Under the null hypothesis of no association at any SNP locus, the points would be expected to follow the slope lines. Deviations from the slope lines correspond to loci that deviate from the null hypotheses.

The Q-Q plots for the test statistics of MMRA are shown in Figure 1. From these plots, it is apparent that there is no clear overall systematic bias in most of traits. However, for the traits of CD4+CD8-, CD4+CD8+ and CD8+ T cells, the effects of population stratification caused an overall slightly systematic bias, and the points where the observed statistics of the significant SNPs are higher than the expected *χ2* statistics at the significance level defined by the permutation tests.

## Discussion

T cells are the major cell populations mediating the adaptive arm of the immune system. Several studies on T cell subpopulations showed that variations in CD4 and CD8 T cell levels and the ratio of CD4 to CD8 are significantly heritable [38-40]. Heritability estimates were around 65% for the ratio of CD4 to CD8, 50% for CD4+ counts, and 56% for CD8+ counts [40]. Therefore, as a category of immune-related traits with high heritability, T-cell subpopulations can be potentially implemented to selection for disease resistance and susceptibility in swine breeding. The present results clearly show that a number of loci contribute to the variation of T-cell subpopulations in peripheral blood in pig. These findings would enhance our understanding of genetic control of the variations of T-cell subpopulations.

In this study, we treated breed as a fixed effect to avoid potential confounding between effects of SNP and breed. Hence, to check whether significant SNPs actually were segregating in the different breeds and showing the same effects in the different breeds, we tested all the significant SNPs based on the improved model considering the interaction of SNP by breed. Finally, no SNP has a significant interaction effect was detected. And the main purpose of our study is to detect common SNPs influencing the T-cell subpopulations in swine, so we did not put the interaction effect in our association model in GWAS.

In this study, we carried out GWAS to explore potential causal genes for the T-cell subpopulations in swine. To our knowledge, this is the first study aiming to reveal the genetic mechanism of those immune traits in swine based on a high density SNP chip panel. Our results revealed 64 significant SNPs associated with the seven traits. Among these identified SNPs, 27 fall into previously reported immune-related QTL regions [27,41,42], including 10 significant SNPs in the regions which have been reported to harbor QTL for lymphocyte previously [27,42-44]. In particular, the significant SNP (ASGA0077977) with effect on the CD4+CD8+ on SSC17 is located within the reported QTL for CD4+CD8+ region [27]; The significant SNP (ASGA0000475) for CD4+CD8+ on SSC1 is located within the reported QTL for CD4+/CD8+ [27]; The significant SNP (ALGA0027442) for CD4-CD8- on SSC4 is located within the reported QTL for CD4+ [27]; The significant SNP for CD4+ on SSC5 (ALGA0030335) fell into the region which has been reported to harbor QTL for CD4+ in our previous studies [27].

Several significant SNPs were found associated with more than one trait in this study. Specifically, SNP H3GA0041012 on SSC14 was associated with CD4+/CD8+, CD4+CD8- and CD4-CD8+ T cells. SNPs ALGA022248 on SSC16 and INRA0055474, ASGA0079256 and ALGA0098112 on SSC18 were associated with two traits, respectively. The traits of T-cell subpopulations are correlated, so a change in one trait may result in a change also in other traits, which should be the reason that these SNP significantly associated with more than one trait.

**Figure 2:**
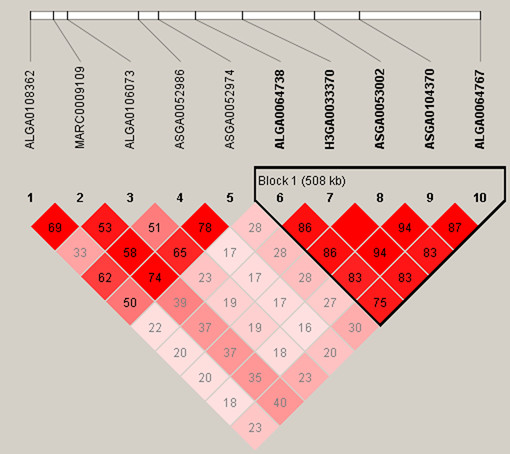
**Linkage disequilibrium (LD) pattern for significant SNPs on SSC12.** LD blocks are marked with triangles. Values in boxes are LD (*r²*) between SNP pairs and the boxes are coloured according to the standard Haploview colour scheme: LOD > 2 and D' = 1, red; LOD > 2 and D' < 1, shades of pink/red; LOD < 2 and D' = 1, blue; LOD < 2 and D' < 1, white (LOD is the log of the likelihood odds ratio, a measure of confidence in the value of D'.

Several regions harboured more than one significant SNP. Six SNPs which have significantly associated with the proportion of CD4+CD8+ T cells fell in the region of 116.7 Mb to 118.1 Mb on SSC13. Ten SNPs which fell in the region of 6.6-7.4 Mb on SSC12 were significantly associated with the proportion of CD4+CD8- T cells. In this region, QTLs for lymphocyte were mapped previously [42]. Additionally, the LD block analysis for SSC12 showed that a LD block exists between ALGA0064738 and ALGA006476, where the genome-wise significant SNP H3GA0033370 was included (Figure 2). 

Several significant SNPs fell into the regions which harbour a number of known immunity-related genes. On SSC12, ten SNPs (one of them with genome-wise significance level) for the proportion of CD4+CD8- T cells fell in the 0.8 Mb region, which harbours *SOX9* ((sex determining region Y)-box 9) gene. This gene has important role in chondrogenesis, sex determination, pigmentation, organ maintenance and cancer. To our knowledge, differential *Sox9* expression is critical for the establishment and maintenance of a regular thymic microenvironment, where specialized stromal cells promote thymocytes development and selection to functionally mature T cells. And the LD block analysis for this region indicated that *SOX9* gene was in LD with the most significant SNP of CD4+CD8- T%, so it has a great potential value of investigation its function on T-cell subpopulations in further research. SNPs ALGA0105115 and MARC0076632 with effects on the CD4+CD8- T cells were found in the region which harbours *CD34* (CD34 molecule) gene. SNPs ASGA0046812 and ALGA0057450 with effects on the CD4+CD8- T cells were found in the region which harbours *AKT3* (v-akt murine thymoma viral oncogene homolog 3 (protein kinase B, gamma)) gene. The *AKT* gene family has been implicated in signal transmission leading to activation, differentiation as well as cellular survival of T-lymphocytes. The candidate genes discussed there need further research to confirm the genetic mechanism on the traits in this study.

## Conclusions

Summary, our study revealed 64 SNPs associated with T-cell subpopulation in peripheral blood in pigs at chromosome-wise significance level (including 3 SNPs at genome-wise significance level) and 27 significance SNPs were located within the immune-related QTL regions reported in previous studies. Furthermore, 14 significant SNPs fell into the regions harboring known immunity-related genes. Findings herein lay a preliminary foundation for further identifying the causal mutations affecting swine immune capacity in follow-up studies.

## **Competing interests**

The authors declare that they have no competing interests.

## **Authors' contributions**

XL and WXF are the major executive persons of all jobs of this study, including collection of the phenotypes, SNP genotyping, statistical analysis, and drafting this manuscript. YRL and YL assisted in phenotype collecting and SNP genotyping. XDD and JPZ assisted in the statistical analysis. JFL and QZ planed and supervised the whole study. All authors read and approved the final manuscript.

## Supplementary Material

Additional file 1 TableS1 Distributions of SNPs after quality control on each chromosome. Click here for file

Additional file 2 FigureS1 **Manhattan plots of GWA for T-cell subpopulations.** Different chromosomes are represented by different colors. Chromosome 19 stands for the X chromosome of swine. Click here for file
