# Supplementary material for: Genome-wide association study for T lymphocyte subpopulations in swine
Source: BMC Genomics. 2012 Sep 18;13:488. doi: 10.1186/1471-2164-13-488 (PMC3481476; doi:10.1186/1471-2164-13-488)
Supplement: Additional file 1 TableS1 — Distributions of SNPs after quality control on each chromosome. [file 1471-2164-13-488-S1.doc]

Supplementary Table Distributions of SNPs after quality control on each chromosome.

| SSC. | No. SNP | Average distance (kb)^a^ |
| --- | --- | --- |
| 1 | 4022 | 73.46 |
| 2 | 2240 | 62.50 |
| 3 | 1973 | 62.00 |
| 4 | 2505 | 54.41 |
| 5 | 1531 | 65.68 |
| 6 | 2020 | 60.72 |
| 7 | 2278 | 59.79 |
| 8 | 1802 | 66.27 |
| 9 | 2309 | 57.38 |
| 10 | 1147 | 57.23 |
| 11 | 1354 | 58.92 |
| 12 | 1061 | 54.12 |
| 13 | 2375 | 61.12 |
| 14 | 2990 | 49.67 |
| 15 | 1839 | 73.05 |
| 16 | 1339 | 57.71 |
| 17 | 1111 | 57.58 |
| 18 | 909 | 59.53 |
| X | 804 | 156.36 |
| 0^b^ | 5224 | NA |
| TOTAL | 40833 |  |

**^a^** Derived from the most recent porcine genome sequence assembly (Sscrofa9.2) (http://www.ncbi.nlm.nih.gov/projects/mapview/map_search.cgi?taxid=9823&build=previous)

**^b^** These SNPs are not assigned to any chromosomes
