# Supplementary material for: Genome-wide association study for T lymphocyte subpopulations in swine
Source: BMC Genomics. 2012 Sep 18;13:488. doi: 10.1186/1471-2164-13-488 (PMC3481476; doi:10.1186/1471-2164-13-488)
Supplement: Additional file 2 FigureS1 — Manhattan plots of GWA for T-cell subpopulations. Different chromosomes are represented by different colors. Chromosome 19 stands for the X chromosome of swine. [file 1471-2164-13-488-S2.doc]

Supplementary Fig.

Manhattan plots of GWA for T-cell subpopulations Different chromosomes are represented by different colors. Chromosome 19 stands for the X chromosome of swine.
